# AMPK activation can delay aging

**DOI:** 10.15190/d.2015.45

**Published:** 2015-12-31

**Authors:** Andreea L. Stancu

**Affiliations:** Department of Pathology, Harvard Medical School and Beth Israel Deaconess Medical Center, Boston, MA, USA

**Keywords:** AMP-activated protein kinase, lifespan, energetic metabolism

## Abstract

AMPK controls the regulation of cellular homeostasis, metabolism, resistance to stress, cell survival and growth, cell death, autophagy, which are some of the most critical determinants of aging and lifespan. Specific AMPK activation was recently shown to delay aging and prolong lifespan in Drosophila melanogaster. Indirect AMPK activators, such as resveratrol, metformin and exercise, are currently in clinical trials for studying their impact on human aging-related characteristics, tissue homeostasis and metabolic dysfunctions. In this minireview, I am briefly discussing the recent advances on AMP involvement in aging and lifespan elongation.

## Introduction

As a sensor of cellular energy status, AMP-activated protein kinase (AMPK) is expressed in almost all eukaryotic cells^1^. Activation of AMPK is able to restore the energy balance when the energy state of a cell is decreased. This is performed by stimulating catabolic processes that generate ATP and by inhibiting anabolic processes that consume ATP^2^. AMPK controls the regulation of cellular homeostasis, metabolism, resistance to stress, cell survival and growth, cell death, autophagy**which are some of the most critical determinants of aging and lifespan^3^. AMPK can integrate critical cellular signals and controls many signaling pathways that regulate these processes. Recent studies show that the AMPK activation and AMPK responsiveness decrease with age, which may explain the altered metabolic regulation, resulting in reduced autophagic clearance of unnecessary products and an increase in oxidative stress^3^.**

Caloric restriction was shown to protect against senescence by increasing autophagic activity and reducing oxidative damage^4^. These mechanisms are at least in part mediated by caloric restriction-induced activation of AMPK and its downstream signaling pathways^5^. Dietary restriction can at least in part mediate longevity by activating the AMPK-FOXO axis^6-8^.

At least five clinical trials (**Table 1**) with AMPK activators, such as resveratrol^9-11^, metformin^12^ and exercise^13,14^, investigate their impact on human aging related characteristics, tissue homeostasis and metabolic dysfunctions^15^.

**Table 1:** Clinical trials investigating the impact of AMPK activators (such as resveratrol, metformin and exercise) on human aging-related characteristics, tissue homeostasis and metabolic dysfunctions

Study name	Status (Dec. 2015)	Conditions	Purpose	Ref.
**Beneficial Effects of Exercise and Healthy Diets on Muscle and Adipose Tissue**	Recruiting	Metabolic Syndrome	Intends to verify if physical exercise and/or Mediterranean diet, in middle aged individuals with metabolic syndrome, preserve adequate adipose tissue functionality and delay skeletal muscle aging; AMPK is one of the studied markers	^16^
**Effect of Age on Glucose and Lipid Metabolism**	Active, not recruiting	Metabolism Disorders	Tests the hypotheses that the decrease in muscle fat oxidation from elderly human individual is secondary to an age-mediated reduction in AMPK signaling, in vivo, and exercise-induced increase in the AMPK signaling will result in/correlate with improved insulin action, increased fat oxidation & reduced intramyocellular lipids	^17^
**Metformin and Longevity Genes in Prediabetes**	Completed	Aging, Insulin Resistance, Prediabetes, Inflammation	Investigates the effects of the AMPK pathway activation on longevity genes and inflammation in the setting of pre-diabetes in vivo and in vitro	^18^
**Metformin in Longevity Study (MILES)**	Ongoing	Aging	Examine the effects of metformin treatment on the potential of changing the gene expression profile of older adults with impaired glucose tolerance, to that of young healthy subjects	^19^
**Resveratrol to Enhance Vitality and Vigor in Elders (REVIVE)**	Not yet recruiting	Physical and Mitochondrial function	Investigates if resveratrol improves the mitochondrial function within the skeletal muscles of elders; AMPK is one of the studied markers	^20^

## AMPK activation protects against aging and elongates lifespan in several species

Recent reports demonstrated that AMPK can exert pro-longevity effects in several species^21^*. *AMPK activation in gastrointestinal tract increases Drosophila melanogaster’s lifespan by 30%, from six weeks to eight weeks^21^. Caloric restriction-induced AMPK activation protects against senescence by increasing autophagic activity and reducing oxidative damage in rats^4^. This process is at least in part executed by the AMPK-FOXO signaling pathway, as shown in C. elegans^6^. Moreover, metformin was shown to prevent sedentariness-related damages in mice, a process which may be related to AMPK activation. In mice, metformin activated signaling mediated by AMPK and CAMKII, while inactivating ERK, thus modulating hepatic stress. In mouse skeletal muscle, metformin induced phosphorylation of Akt and its activation, process important for skeletal muscle mass maintenance^22^.

## AMPK signaling and aging

AMPK is shown to be a central regulator and integrator for several intracellular signaling pathways controlling cellular homeostasis, metabolism, response to stress, oxidative damage proliferation, cell growth, cell death, autophagy, cellular polarity and cellular senescence. Some of these pathways were shown to promote longevity in lower organisms. It is now well known that DAF-16/FoxO transcription factor can be regulated by AMPK^23^ and acts as a pro-longevity axis in C. elegans^3^. FoxO family of transcription factors are well known for the regulation a broad range of biological critical processes, such as apoptosis, cell cycle progression, resistance to oxidative stress, metabolism, differentiation and senescence^23-25^. Moreover, muscle aging can be delayed by modulating the muscle-specific dFOXO/4E-BP/activin signaling, which can induce autophagy. These events are related to extended organismal lifespan^26,27^.

Other groups have reported the involvement of p53 tumor suppressor, NF-kB signaling pathway and Sirtuins in cellular senescence and aging of mammalian organisms. SIRT1 is well known for inducing signaling changes that mediate caloric restriction-induced lifespan elongation^2,28^. Several other AMPK downstream signaling pathways with potential involvement in aging were previously described^3^.

## Targeting AMPK activation to increase lifespan

Discovery of AMPK’s critical cellular functions has led to the identification of a huge number of products that can (most of them indirectly) activate AMPK. To date, over 100 natural products (many used in Asian medicine) are uncovered. Very few of them directly modulate AMPK, however, even those are expected to have AMPK-independent effects^1^. For example, salicylate is shown to directly bind AMPK, although it also binds and modulates the activity of other cellular enzymes. Some of these compounds, indirectly activate AMPK by inhibiting mitochondrial respiratory chain: berberine, galegine etc. Moreover, at least two of these compounds, salicylate and metformine, are some of the most used drugs worldwide for the treatment of common pathologies. Although these drugs can activate AMPK, involvement of AMPK in their therapeutic effects is not yet well characterized^1^. Metotrexate was also recently shown to activate AMPK, promoting glucose uptake and lipid oxidation in skeletal muscle^27^.

In 2015, the US Food and Drug Administration (FDA) has given the green light for human clinical trials evaluating the potential metformin-induced elongation of human lifespan^19^. In addition to its well characterized effects of regulating the glucose metabolism, being used in the treatment of type 2 diabetes for many years, metformin can influence a wide rage of cellular processes critical in aging process and the development of age-related conditions, such as apoptosis, autophagy, cellular senescence, oxidative damage and inflammation^28-31^. Interestingly, metformin mimics some of the benefits of calorie restriction without a decrease in caloric intake. It improves physical performance, reduces cholesterol and low-density lipoprotein levels and increases sensitivity to insulin^32^. Both metformin and rapamycin can prolong lifespan in mice^32,33^. AMPK was previously shown to mediate the anti-aging effects of metformin^32^, while autophagy was proposed to be involved in inducing the anti-aging effects of rapamycin^34,35^.

## Conclusions

AMPK is a sensor of cellular energy status and a critical regulator of cellular homeostasis, metabolism response to stress, oxidative damage and many other processes involved in aging. We now know, that localized activation of AMPK in key tissues such as the brain, can slow aging in a non-cell autonomous manner^21^*. *AMPK activation in the Drosophila’s nervous system induces autophagy both in the brain and the intestinal epithelium, which is related to the anti-aging effects and extended lifespan^21^. Autophagy, which is a bulk protein degradation process^35,36^, was previously proposed to be involved in inducing the anti-aging effects of rapamycin^34,35^. Thus, AMPK-induced autophagic clearance and increased resistance to stress are major players involved in lifespan elongation in lower organisms. Recent reports^3,21,37^ established that AMPK activation and AMPK responsiveness decrease with age, which may explain the altered metabolic regulation, resulting in reduced autophagic clearance of unnecessary products (via mTOR), an increase in oxidative stress and decrease resistance to cellular stress (potentially due to DAF-16/FoxO and/or p53 signaling pathways downregulation). Thus, finding efficient strategies of increasing AMPK responsiveness and activation may be of important use as anti-aging treatments and for lifespan elongation. Metformin, resveratrol and exercise are the leading examples currently tested in human clinical trials.

- **AMPK activation/responsiveness decreases with age, resulting in**:
  - *reduced autophagic clearance of unnecessary products *
  - *an increase in oxidative stress *
  - *a decrease resistance to cellular stress *
- **AMPK-mediated autophagic clearance and increased resistance to stress are major players involved in lifespan extension in lower organisms**.

## References

[1] Grahame Hardie David (2016). Regulation of AMP-activated protein kinase by natural and synthetic activators.. Acta pharmaceutica Sinica. B.

[2] Ruderman Neil B., Julia Xu X., Nelson Lauren, Cacicedo José M., Saha Asish K., Lan Fan, Ido Yasuo (2010). AMPK and SIRT1: a long-standing partnership?. American Journal of Physiology-Endocrinology and Metabolism.

[3] Salminen Antero, Kaarniranta Kai (2012). AMP-activated protein kinase (AMPK) controls the aging process via an integrated signaling network. Ageing Research Reviews.

[4] Ning Yi-Chun, Cai Guang-Yan, Zhuo Li, Gao Jian-Jun, Dong Dan, Cui Shaoyuan, Feng Zhe, Shi Suo-Zhu, Bai Xue-Yuan, Sun Xue-Feng, Chen Xiang-Mei (2013). Short-term calorie restriction protects against renal senescence of aged rats by increasing autophagic activity and reducing oxidative damage. Mechanisms of Ageing and Development.

[5] García-Prieto C.F., Pulido-Olmo H., Ruiz-Hurtado G., Gil-Ortega M., Aranguez I., Rubio M.A., Ruiz-Gayo M., Somoza B., Fernández-Alfonso M.S. (2015). Mild caloric restriction reduces blood pressure and activates endothelial AMPK-PI3K-Akt-eNOS pathway in obese Zucker rats. Vascular Pharmacology.

[6] Greer Eric L, Dowlatshahi Dara, Banko Max R, Villen Judit, Hoang Kimmi, Blanchard Daniel, Gygi Steven P, Brunet Anne (2007). An AMPK-FOXO pathway mediates longevity induced by a novel method of dietary restriction in C. elegans.. Current biology : CB.

[7] Greer Eric L, Banko Max R, Brunet Anne (2009). AMP-activated protein kinase and FoxO transcription factors in dietary restriction-induced longevity.. Annals of the New York Academy of Sciences.

[8] Greer Eric L, Brunet Anne (2005). FOXO transcription factors at the interface between longevity and tumor suppression.. Oncogene.

[9] Chen Sifan, Zhou Niman, Zhang Zili, Li Wenxue, Zhu Wei (2015). Resveratrol induces cell apoptosis in adipocytes via AMPK activation.. Biochemical and biophysical research communications.

[10] Chen Sifan, Li Zilun, Li Wenxue, Shan Zhiming, Zhu Wei (2011). Resveratrol inhibits cell differentiation in 3T3-L1 adipocytes via activation of AMPK.. Canadian journal of physiology and pharmacology.

[11] Chen Sifan, Xiao Xincai, Feng Xiang, Li Wenxue, Zhou Niman, Zheng Lin, Sun Yanshuang, Zhang Zili, Zhu Wei (2012). Resveratrol induces Sirt1-dependent apoptosis in 3T3-L1 preadipocytes by activating AMPK and suppressing AKT activity and survivin expression.. The Journal of nutritional biochemistry.

[12] Duca Frank A, Côté Clémence D, Rasmussen Brittany A, Zadeh-Tahmasebi Melika, Rutter Guy A, Filippi Beatrice M, Lam Tony K T (2015). Metformin activates a duodenal Ampk-dependent pathway to lower hepatic glucose production in rats.. Nature medicine.

[13] Fentz Joachim, Kjøbsted Rasmus, Kristensen Caroline Maag, Hingst Janne Rasmus, Birk Jesper Bratz, Gudiksen Anders, Foretz Marc, Schjerling Peter, Viollet Benoit, Pilegaard Henriette, Wojtaszewski Jørgen F. P. (2015). AMPKα is essential for acute exercise-induced gene responses but not for exercise training-induced adaptations in mouse skeletal muscle. American Journal of Physiology-Endocrinology and Metabolism.

[14] Lundberg Tommy R., Fernandez-Gonzalo Rodrigo, Tesch Per A. (2014). Exercise-induced AMPK activation does not interfere with muscle hypertrophy in response to resistance training in men. Journal of Applied Physiology.

[15] (2015). https://clinicaltrials.gov/ct2/results?term=AMPK%2
C+aging&Search=Search. ClinicalTrials.gov; accessed in December 2015.

[16] (2015). ClinicalTrials.gov registry of U.S. NIH; NCT01793896; 
https://clinicaltrials.gov/ct2/show/NCT01793896. ClinicalTrials.gov; accessed in December 2015.

[17] (2015). ClinicalTrials.gov registry of U.S. NIH; NCT01737164;
https://clinicaltrials.gov/ct2/show/NCT01737164. ClinicalTrials.gov; accessed in December 2015.

[18] (2015). ClinicalTrials.gov registry of U.S. NIH; NCT01765946; https://clinicaltrials.gov/ct2/show/NCT01765946. ClinicalTrials.gov; accessed in December 2015.

[19] (2015). ClinicalTrials.gov registry of U.S. NIH; NCT02432287;
https://clinicaltrials.gov/ct2/show/NCT02432287. ClinicalTrials.gov; accessed in December 2015.

[20] (2015). ClinicalTrials.gov registry of U.S. NIH; NCT02123121;
https://clinicaltrials.gov/ct2/show/NCT02123121. ClinicalTrials.gov; accessed in December 2015.

[21] Ulgherait Matthew, Rana Anil, Rera Michael, Graniel Jacqueline, Walker David W (2014). AMPK modulates tissue and organismal aging in a non-cell-autonomous manner.. Cell reports.

[22] Senesi Pamela, Montesano Anna, Luzi Livio, Codella Roberto, Benedini Stefano, Terruzzi Ileana (2016). Metformin Treatment Prevents Sedentariness Related Damages in Mice.. Journal of diabetes research.

[23] Dumitrascu Georgiana R, Bucur Octavian (2013). Critical physiological and pathological functions of Forkhead Box O tumor suppressors. Discoveries.

[24] Plati Jessica, Bucur Octavian, Khosravi-Far Roya (2008). Dysregulation of apoptotic signaling in cancer: molecular mechanisms and therapeutic opportunities.. Journal of cellular biochemistry.

[25] Singh Amrik, Ye Min, Bucur Octavian, Zhu Shudong, Tanya Santos Maria, Rabinovitz Isaac, Wei Wenyi, Gao Daming, Hahn William C, Khosravi-Far Roya (2010). Protein phosphatase 2A reactivates FOXO3a through a dynamic interplay with 14-3-3 and AKT.. Molecular biology of the cell.

[26] Wang Yu, Liang Yan, Vanhoutte Paul M (2011). SIRT1 and AMPK in regulating mammalian senescence: a critical review and a working model.. FEBS letters.

[27] Pirkmajer Sergej, Kulkarni Sameer S, Tom Robby Z, Ross Fiona A, Hawley Simon A, Hardie D Grahame, Zierath Juleen R, Chibalin Alexander V (2015). Methotrexate promotes glucose uptake and lipid oxidation in skeletal muscle via AMPK activation.. Diabetes.

[28] Anwar M Akhtar, Kheir Wassim Abou, Eid Stephanie, Fares Joanna, Liu Xiaoqi, Eid Ali H, Eid Assaad A (2014). Colorectal and Prostate Cancer Risk in Diabetes: Metformin, an Actor behind the Scene.. Journal of Cancer.

[29] Bhat Aparna, Sebastiani Giada, Bhat Mamatha (2015). Systematic review: Preventive and therapeutic applications of metformin in liver disease.. World journal of hepatology.

[30] Na Hyun-Jin, Park Joung-Sun, Pyo Jung-Hoon, Jeon Ho-Jun, Kim Young-Shin, Arking Robert, Yoo Mi-Ae (2015). Metformin inhibits age-related centrosome amplification in Drosophila midgut stem cells through AKT/TOR pathway.. Mechanisms of ageing and development.

[31] Na Hyun-Jin, Park Joung-Sun, Pyo Jung-Hoon, Lee Shin-Hae, Jeon Ho-Jun, Kim Young-Shin, Yoo Mi-Ae (2013). Mechanism of metformin: inhibition of DNA damage and proliferative activity in Drosophila midgut stem cell.. Mechanisms of ageing and development.

[32] Martin-Montalvo Alejandro, Mercken Evi M, Mitchell Sarah J, Palacios Hector H, Mote Patricia L, Scheibye-Knudsen Morten, Gomes Ana P, Ward Theresa M, Minor Robin K, Blouin Marie-José, Schwab Matthias, Pollak Michael, Zhang Yongqing, Yu Yinbing, Becker Kevin G, Bohr Vilhelm A, Ingram Donald K, Sinclair David A, Wolf Norman S, Spindler Stephen R, Bernier Michel, de Cabo Rafael (2013). Metformin improves healthspan and lifespan in mice.. Nature communications.

[33] Harrison David E, Strong Randy, Sharp Zelton Dave, Nelson James F, Astle Clinton M, Flurkey Kevin, Nadon Nancy L, Wilkinson J Erby, Frenkel Krystyna, Carter Christy S, Pahor Marco, Javors Martin A, Fernandez Elizabeth, Miller Richard A (2009). Rapamycin fed late in life extends lifespan in genetically heterogeneous mice.. Nature.

[34] Bjedov Ivana, Toivonen Janne M, Kerr Fiona, Slack Cathy, Jacobson Jake, Foley Andrea, Partridge Linda (2010). Mechanisms of life span extension by rapamycin in the fruit fly Drosophila melanogaster.. Cell metabolism.

[35] Rubinsztein David C, Mariño Guillermo, Kroemer Guido (2011). Autophagy and aging.. Cell.

[36] Bucur Octavian, Ray Subrata, Bucur Maria Cristina, Almasan Alexandru (2006). APO2 ligand/tumor necrosis factor-related apoptosis-inducing ligand in prostate cancer therapy.. Frontiers in bioscience : a journal and virtual library.

[37] Burkewitz Kristopher, Zhang Yue, Mair William B (2014). AMPK at the nexus of energetics and aging.. Cell metabolism.

